# Allergic contact dermatitis by shampoo components: a descriptive analysis of 20 cases^☆^^☆☆^

**DOI:** 10.1016/j.abd.2019.12.009

**Published:** 2020-07-04

**Authors:** Rosana Lazzarini, Lilian Lemos Costa, Nathalie Mie Suzuki, Mariana de Figueiredo Silva Hafner

**Affiliations:** a Allergy Unit, Dermatology Clinic, Santa Casa de Misericórdia de São Paulo, São Paulo, SP, Brazil; b Dermatology Clinic, Santa Casa de Misericórdia de São Paulo, São Paulo, SP, Brazil

Dear Editor,

Shampoos are the most used cosmetics for hair and scalp care. They are composed of several ingredients, usually ranging from 10 to 30, with different functions, such as surfactants for cleanning, preservatives to stabilize the product, and fragrances to make them cosmetically pleasant to the consumer.^1^, ^2^, ^3^

Adverse reactions to their use may occur. Although studies demonstrate that the sensitizing power of allergens present in rinse products is low (through ephemeral contact with the skin), allergic contact dermatitis (ACD) due to components of shampoos has been well described in the literature.^4^ It is possible that sensitization occurs earlier by contact with other products containing the same allergens. In addition, factors such as frequency of use and presence of atopy may influence the onset of ACD.

Pruritus and hair loss are the most described symptoms. Eczematous lesions are usually observed on the scalp, face, ears, and cervical region.^1^, ^5^ In these cases, the patch test is the main tool for the identification of the causal agent and, subsequently, treatment.

This study aimed to evaluate the main allergens that cause ACD by shampoos and the epidemiological characteristics of the population affected by this condition in a specialized dermatological service of a quaternary hospital.

A total of 654 patch tests were carried out between January 2014 and August 2019. Among them, those with a final diagnosis of ACD by shampoo were chosen for analysis. All selected cases were tested with the Brazilian standard (FDA Allergenic, Brazil), capillary (IPI ASAC, Brazil), and Latin American (Chemothecnique, Sweden) tests.

ACD by shampoo was diagnosed in 20 patients (3% of those who underwent the patch test). Of these, 19 (95%) were female and one male. The mean age was 52.2 years. The higher frequency of female patients is consistent with the greater use of cosmetics by this group.

The mean time of illness was 46 months, reflecting probable difficulties in establishing the diagnosis, thus lengthening the time of illness since the causative agents were not withdrawn.

The most affected regions in patients were: scalp in 12 cases (60%), face and upper limbs in ten (50%) each, cervical in seven (35%), back in four (20%), ears in three (15%), chest and armpits in two (10%) each, and abdomen and shoulders in one (5%) each; these data are compatible with the literature. This variety of possible clinical presentations contributes to diagnostic difficulties, particularly when there are no evident lesions on the scalp, as occurred in eight out of 20 patients (40%), which can be explained by the anatomical characteristics of this region (great thickness and greater number of pilosebaceous units), which hinder the penetration of allergens and the detection of eczema.^1^, ^2^ In some patients, the lesions affected the areas that come into contact with the shampoo when it is rinsed: the forehead, eyelids, auricular region, lateral cervical, and back (Figure 1, Figure 2, Figure 3).

**Figure 1:**
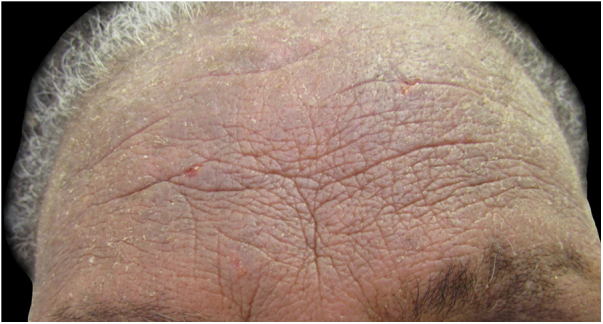
Patient with chronic eczema (intense lichenification on the forehead) due to allergic contact dermatitis from methyl isothiazolinone present in the shampoos used.

**Figure 2:**
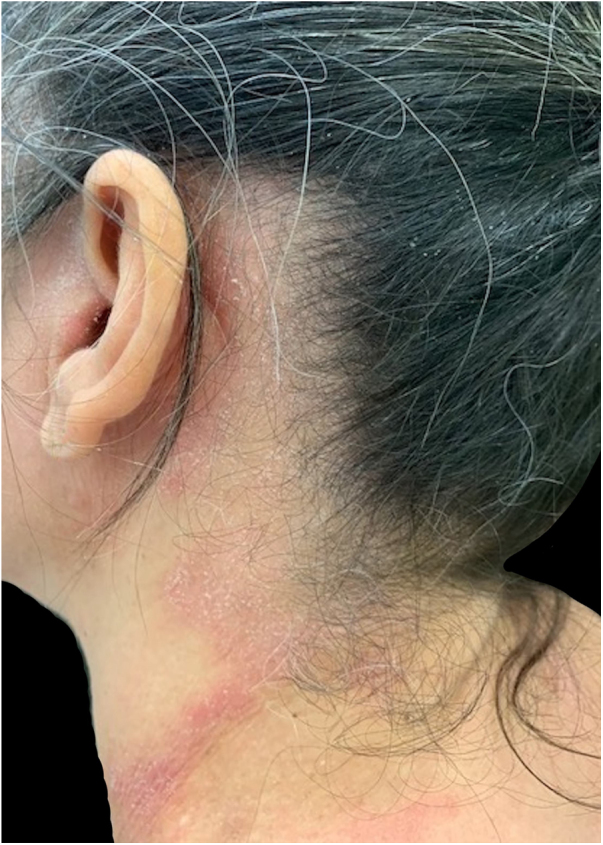
Patient with allergic contact dermatitis to components of the shampoos involving the pre-auricular, retroauricular, and lateral cervical regions (areas that come into contact with the shampoo when it is rinsed).

**Figure 3:**
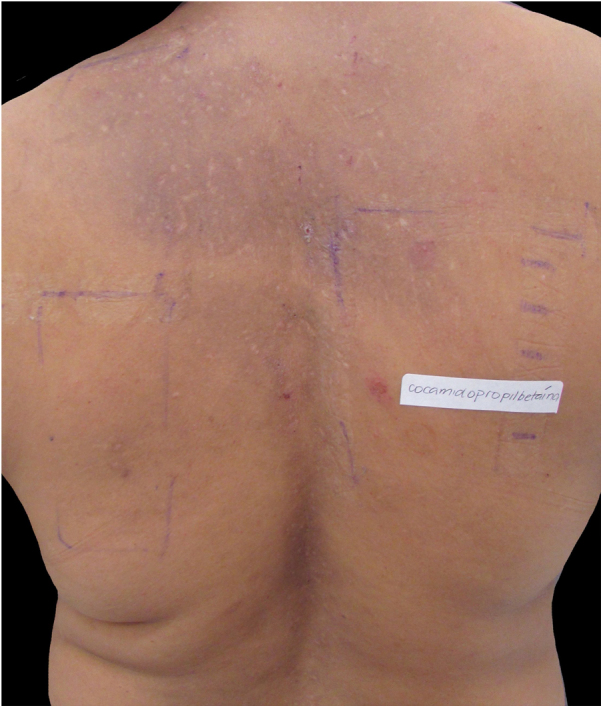
Patient with hyperchromia and abrasions on the back caused by pruritus from allergic contact dermatitis to cocamidopropyl betaine in shampoos (demonstrated by patch test).

The relevant positive results of the patch tests are shown in Table 1. The responsible allergens were preservatives (Kathon CG, formaldehyde, captan, methyldibromo glutaronitrile and dyazolinidyl urea), fragrances (FM1, FM2, and balsam of Peru), and surfactants (cocamidopropyl betaine, lauryl polyglucoside, and decyl glucoside). These results are in agreement with the literature.^1^ It is noteworthy that among the eleven ACD-causing allergens presented, only four are included in the Brazilian standard test (Kathon CG, formaldehyde, fragrance-mix 1, and balsam of Peru), while the others were present in the complementary tests used. In all cases, the diagnosis was confirmed by a test with current relevance, verified by reading the labels of the shampoos used (proving the exposure to the detected allergens), and the complete improvement after removal of these agents during clinical follow-up.

**Table 1:** Relevant allergens found in patch tests.

Relevant allergens	Number of positive tests	%
Kathon CG^a^	12	26.0
Formaldehyde	8	17.0
Cocamidopropyl betaine	5	11.0
Captan^b^	5	11.0
Lauryl polyglucoside	4	8.5
Decyl glucoside	4	8.5
Methyldibromo glutaronitrile	3	6.0
FM1^c^	2	4.0
FM2^d^	2	4.0
Diazolinidyl urea	1	2.0
Balsam of Peru	1	2.0
Total	47^e^	100.0

a Kathon CG: methylisothiazolinone + methylchloroisothiazolinone.

b Captan: N-trichloromethylthio-4-cyclohexene-1,2-dicarboximide.

c FM1: geraniol, cinnamaldehyde, hydroxycitronellal, cinnamyl alcohol, amylcinnamaldehyde, isoeugenol, eugenol, and oak moss.

d FM2: coumarin, lyral, citronellol, farnesol, citral, hexyl cinnamic aldehyde.

e NOTE: some patients presented more than one positive test.

As most shampoos have similar compositions, it is common for the dermatitis to persist even after patients change the product and brand on their own. Thus, it is essential to perform a patch test whenever there is clinical suspicion, which should be performed with the standard and the complementary series, thus allowing individualized guidance for each patient regarding which products should be used.

## Financial support

None declared.

## Authors’ contributions

Rosana Lazzarini: Approval of the final version of the manuscript; conception and planning of the study; effective participation in research orientation.

Lilian Lemos Costa: Obtaining, analyzing, and interpreting the data; critical review of the literature.

Nathalie Mie Suzuki: Elaboration and writing of the manuscript; critical review of the manuscript.

Mariana de Figueiredo Silva Hafner: Conception and planning of the study; elaboration and writing of the manuscript; critical review of the manuscript.

## Conflicts of interest

None declared.
